# The Role of Pancreatic Fatty Acid Synthesis in Islet Morphology and Function after Caloric Restriction or Roux-En-Y Gastric Bypass Surgery in Mice

**DOI:** 10.3390/genes14010005

**Published:** 2022-12-20

**Authors:** Haocong Mo, Yang Liu, Mengyuan Zhang, Zirui Qiu, Yilin Li, Zhejiao Zhang, Yanting Li, Geyang Xu

**Affiliations:** 1 Department of Physiology, School of Medicine, Jinan University, 601 Huangpu Avenue West, Guangzhou 510632, China; 2 Center for Clinical Epidemiology and Methodology (CCEM), Guangdong Second Provincial General Hospital, Guangzhou 510317, China

**Keywords:** Roux-en-Y gastric bypass, caloric restriction, diabetes, apoptosis, fatty acid synthesis, β-cell

## Abstract

Background: Both caloric restriction (CR) and Roux-en-Y gastric bypass (RYGB) are practical interventions for type 2 diabetes mellitus (T2DM), while the molecular mechanisms of CR and RYGB regarding glycemic control are still poorly understood. Here, we explore the effects and underlying mechanisms of CR and RYGB on β-cell area and function. Methods: Average islet size was measured by histological analysis. The pancreatic lipid content was detected by using a commercial lipid assay kit. The expression levels of lipogenic transcription factors and enzymes in mouse pancreas were determined by quantitative PCR, Western blot, and immunofluorescence. Results: CR decreased the mean size of islets and pancreatic insulin production in both regular diet-fed and high-fat diet-fed mice. Increased β-cell apoptosis was detected in the calorie-restricted mice. Interestingly, the lipogenic transcription factors and enzymes such as SREBP1c, PPARγ, FASN and ACC were upregulated in the pancreas after CR. In contrast to CR, RYGB decreased the apoptosis of β-cells and the expression of fatty acid synthase. Conclusions: Pancreatic fatty acid synthesis is critical to the β-cell function after CR and RYGB.

## 1. Introduction

Diabetes rates have reached epidemic proportions [1]. A total of 90% of diabetic patients have T2DM [2]. T2DM are at high risk for neuropathy, retinopathy, nephropathy, and cardiovascular disease [3,4,5,6]. It is characterized by islet dysfunction and β-cell failure [7]. In the early stages, insulin resistance triggers a rise in β-cell mass and function. A continuously excessive β-cell workload progressively contributes to the dysfunction and eventual loss of β-cells [8,9,10]. Oxidative stress, glucotoxicity, lipotoxicity, and islet cell amyloid deposition lead to the loss of β-cells and islet dysfunction [11,12,13,14]. At present, both caloric restriction [15] and bariatric surgery [16] are effective treatments for T2DM. CR, a restriction in caloric intake, has been demonstrated to reverse both β-cell failure and insulin resistance [17,18,19,20]. RYGB, a commonly performed bariatric surgery, is used to treat T2DM. Improvement of islet morphology and function after gastric bypass has been observed in rodents [21,22], pigs [23], and humans [24].

The role of free fatty acid (FFA) in β-cell function is complex and vital. Sufficient circulating FFA is crucial for glucose-stimulated insulin secretion (GSIS) [25]. Transient increases in circulating FFA levels lead to supranormal GSIS [26]. However, studies illustrate that in vitro overexpression of SREBP1c or ACC in islets induces lipid accumulation in β-cells and reduces GSIS [27,28]. A sustained and excessive exposure of β-cell to FFA results in triglyceride (TG) accumulation and impaired GSIS, reduced insulin secretion, and induced the apoptosis and demise of β-cells [12,29,30].

Insulin resistance is a critical factor for the increase in circulating FFA in the development of T2DM. Conversely, elevated circulating FFA contributes to increased β-cell mass and function to compensate for insulin resistance [31]. Due to the importance of endogenous lipids in β-cell function, we hypothesized that pancreatic endogenous lipid synthesis is essential to the islet morphology and function in diet-induced obese diabetic mice (DIO) after CR and RYGB. In the present study, we examine the effects of dietary restriction and gastric bypass on the expression of fatty acid synthesis-related genes in the pancreas and the alterations of β-cell apoptosis in mice.

## 2. Materials and Methods

### 2.1. Materials

Rabbit anti-PPARγ, rabbit anti-ACC antibodies, and mouse anti-β-actin were purchased from Cell Signaling Technology (Beverly, MA, USA). Rabbit anti-fatty acid synthase, anti-death receptor Fas/CD95 antibodies, and mouse anti-SREBP1c were obtained from Abcam Inc. (Cambridge, MA, USA). Rabbit anti-active caspase-3 antibodies, and mouse anti-insulin were obtained from Servicebio (Wuhan, China). Goat anti-mouse FITC-conjugated IgG and DyLight 594 AffiniPure donkey anti-rabbit IgG were obtained from EarthOx LLC (San Francisco, CA, USA). The normal chow diet (D12450B) and the high-fat diet (D12492) were obtained from Research Diets, Inc. (New Brunswick, NJ, USA).

### 2.2. Animals and Treatments

Male C57BL/6J mice were kept in a controlled environment (12 h light-12 h dark cycle, 24 °C). The mice used in the current study were handled in conformity with the Guide for the Care and Use of Laboratory Animals published by the National Institutes of Health (NIH Publications No. 8023, revised 1978). All the protocols have been reviewed and approved by the Animal Care and Use Committee of Jinan University.

To evaluate the effects of CR on pancreatic morphology and function in normal chow diet-fed mice, 8-week-old male mice were randomly assigned to the ad libitum fed (NCD) group or to the group fed with 30% fewer calories than the average daily calories consumed by the ad libitum fed group (NCR). Tissues were harvested after 4 weeks of treatment (*n* = 5 per group).

To test the effects of CR on pancreas in high-fat diet-fed mice, 4-week-old mice were fed a high-fat diet ad libitum (HFD), or 30% less of the mean daily calories consumed by the high-fat diet ad libitum group (HCR) for 8 weeks (six mice per group).

To assess the effects of RYGB on pancreas in diabetic mice, 4-week-old mice were fed a HFD ad libitum for 16 weeks, then the animals were divided into the sham-group (*n* = 11) or the RYGB group (*n* = 9). After one month recovery period, tissues were harvested.

### 2.3. RYGB Procedures

The RYGB- and sham-operations were done as previously described [32].

### 2.4. Intraperitoneal Glucose Tolerance Test (IPGTT)

After 8 h fasting, mice received intraperitoneal injection of glucose at the dose of 1.5 g/kg body weight. Blood sugar levels were assessed at indicated time points after glucose administration with Glucotrend from Roche Diagnostics (Mannheim, Germany).

### 2.5. Measurement of Plasma Insulin

Blood samples were obtained in the presence of EDTA. Insulin was assayed using the enzyme immunoassay kits according to the manufacturer’s instructions.

### 2.6. Histological Analysis

Pancreas tissues were harvested, fixed in 4% paraformaldehyde, paraffin-embedded, cut into sections, and stained with hematoxylin-eosin according to standard procedures. Photomicrographs were taken under an inverted microscope (Leica, Germany). Islet sizes were measured using ImageJ software [33].

### 2.7. Immunohistochemistry

After citrate buffer antigen retrieval, pancreatic sections were blocked and then incubated overnight with rabbit anti-active caspase-3 (1:1000), anti-Fas (1:100), or anti-fatty acid synthase (1:150) combined with mouse anti-insulin (1:300). The tissue sections were then incubated with a mixture of secondary antibodies. Photomicrographs were taken and computerised image analysis was performed to quantify the fluorescence signals.

### 2.8. Determination of Triglycerides in the Pancreas

The triglyceride content in the pancreas tissue was detected according to the manufacturer’s instructions (Boxbio, Beijing, China).

### 2.9. Western Blot Analysis

Proteins were extracted from pancreatic tissues, separated with SDS-PAGE gels, and measured as previously described [32].

### 2.10. RNA Extraction, Quantitative Real-Time PCR

RNA was extracted and reverse-transcribed into cDNAs using RT-PCR kit (Takara). Real-time PCR was performed as previously described [32]. Gene-specific primer pairs used in current study are shown in Table 1.

### 2.11. Statistical Analysis

All the data are expressed as the mean ± S.E.M. Statistical analysis was performed by Student’s *t*-test. The data were considered significant when *p* < 0.05.

## 3. Results

### 3.1. Effects of CR on the Morphology of Islets and Pancreatic Lipogenic Gene Expression in Normal Mice

**Figure 1:**
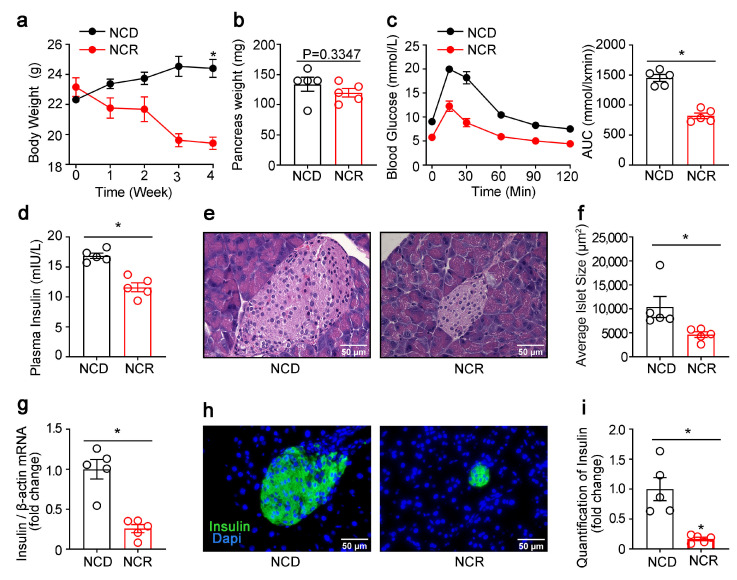
CR reduced β-cell area and insulin production in regular diet-fed mice. (**a**) Body weight curves of calorie-restricted NCD-fed mice. (**b**) Weight of the pancreas. (**c**) Intraperitoneal glucose tolerance test. (**d**) Insulin levels were measured at 15 min after glucose injection. (**e**) HE staining of pancreatic sections. (**f**) Quantification of islet size. (**g**) Insulin mRNA. (**h**) Immunofluorescence staining for insulin (insulin, green) in the pancreases of NCD or a diet restricted to 70% of the calories of the ad libitum group (NCR). Nuclei were stained with DAPI (blue). (**i**) Quantification of insulin. The results were expressed as mean values ± SEM. (Five mice per group) * *p* < 0.05 vs. NCD mice.

CR decreased the body weight but not the pancreas weight significantly (Figure 1a,b). IPGTT showed that NCR mice had lower glycemic excursions in response to i.p. glucose (Figure 1c). Plasma insulin levels (Figure 1d) and the insulin mRNA levels in the pancreas were significantly lower in the NCR group (Figure 1g). H-E staining and immunofluoresence staining of insulin on pancreas tissues indicated decreased average islet size (Figure 1e,f) and β-cell mass (Figure 1h,i) after nutritional intervention.

**Figure 2:**
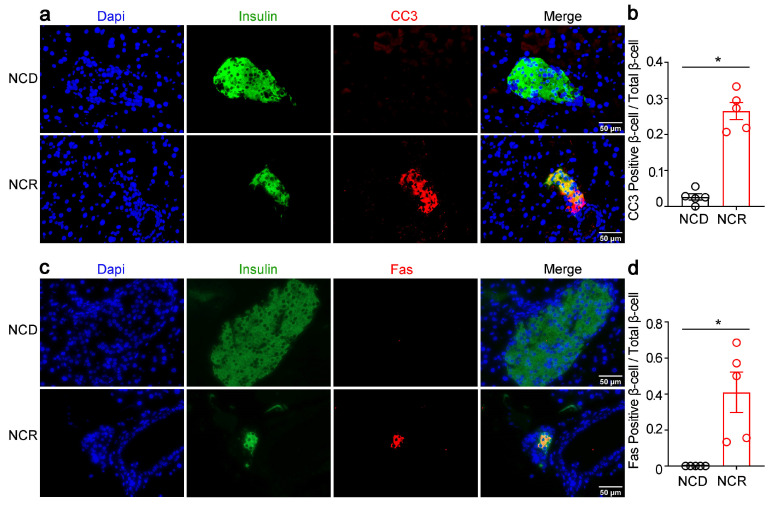
CR stimulated β-cell apoptosis in normal chow diet-fed mice. (**a**,**c**) Representative immunofluorescence images of cleaved caspase-3 (CC3, red), death receptor Fas/CD95 (Fas, red), and insulin (green). (**b**) Quantification of CC3-positive β-cells/total β-cells (β-cell apoptosis frequency). (**d**) Quantification of Fas-positive β-cells/total β-cells. The results were expressed as mean values ± SEM. (Five mice per group) * *p* < 0.05 vs. NCD mice.

We further examined whether CR led to apoptosis in β-cells. We performed double immunofluorescence staining of insulin and cleaved caspase-3 (CC3), as well as insulin and death receptor Fas/CD95 on mouse pancreas tissues. As shown in Figure 2, immunofluorescence signals of CC3 (Figure 2a,b) and Fas/CD95 (Figure 2c,d) were increased in NCR mice islets, which co-localized with the insulin signals, suggesting increased β-cell apoptosis after CR.

**Figure 3:**
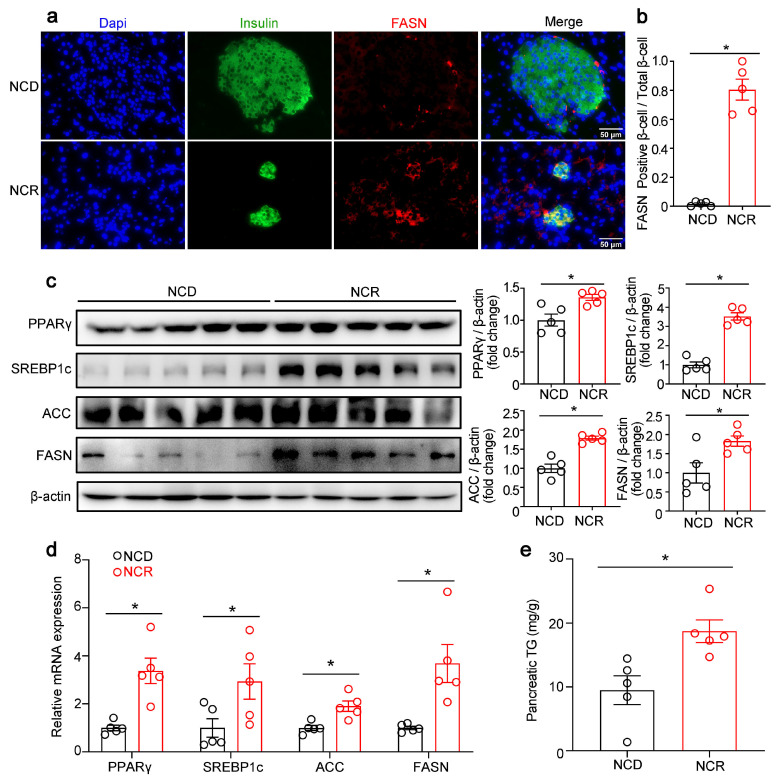
CR increased the lipogenic gene expression in pancreas of normal chow diet-fed mice. (**a**) Representative immunofluorescence images of fatty acid synthase (FASN, red) and insulin (insulin, green). (**b**) Quantification of FASN-positive β-cells/total β-cells. (**c**) Representative Western blot of mouse pancreas. PPARγ, SREBP1c, ACC, and FASN were measured. Quantification of PPARγ, SREBP1c, ACC, and FASN. (**d**) PPARγ, SREBP1c, ACC, and FASN mRNA levels. (**e**) Pancreatic triglyceride. The results were expressed as mean values ± SEM. (*n* = 5 per group) * *p* < 0.05 vs. NCD mice.

Previous studies reported that excessive exposure of β-cell to FFA impaired β-cell function and induced β-cell apoptosis. Therefore, we examined the lipid synthesis pathway in the NCD and NCR groups. Immunofluorescence staining showed that the fatty acid synthase (FASN) signal was increased in the β-cells of NCR mice (Figure 3a,b). Expression levels of lipogenic transcription factors such as SREBP1c and PPARγ and enzymes of lipid synthesis such as FASN and ACC were significantly upregulated in the NCR group (Figure 3a–d). Consistent with the upregulation of the lipid synthesis pathway, increased pancreatic triglyceride content was detected in NCR mice (Figure 3e).

### 3.2. Effects of CR on the Function and Morphology of Pancreatic Islets as well as Lipogenic Gene Expression in Diabetic Mice

**Figure 4:**
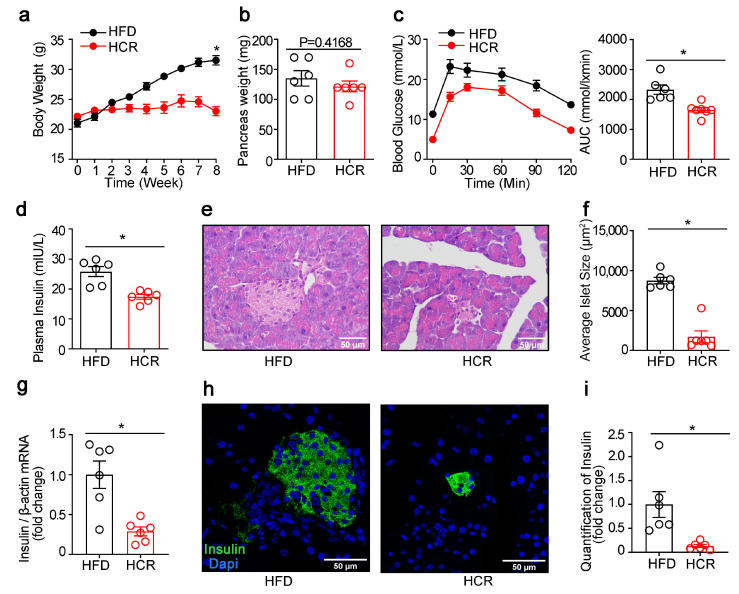
CR decreased insulin production in diabetic mice. (**a**) Body weight curves of CR-treated high-fat diet-fed mice. (**b**) Weight of pancreas. (**c**) Intraperitoneal glucose tolerance test. (**d**) Plasma insulin at 15 min after glucose injection. (**e**) HE staining of pancreatic sections. (**f**) Quantification of islet size. (**g**) Insulin mRNA. (**h**) Immunofluorescence staining for insulin (green) in the pancreas of mice fed with HFD or HCR. (**i**) Quantification of insulin. The results were expressed as mean values ± SEM. (six mice per group), * *p* < 0.05 vs. HFD mice.

**Figure 5:**
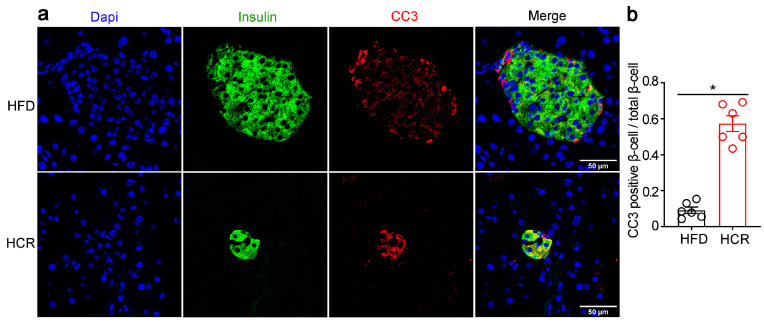
CR induced islet β-cell apoptosis in diabetic mice. (**a**) Representative immunofluorescence images of cleaved caspase-3 (CC3, red) and insulin (green). (**b**) Quantification of CC3-positive β-cells/total β-cells. The results were expressed as mean values ± SEM. (*n* = 6 per group), * *p* < 0.05 vs. HFD mice.

CR led to remarkable body weight losses even when the animals were exposed to a high-fat diet, but had no significant effect on pancreas weight (Figure 4a,b). Simultaneously, the impaired glucose metabolism was significantly improved in HCR mice (Figure 4c). Meanwhile, lower plasma insulin levels (Figure 4d) and insulin mRNA levels (Figure 4g), as well as smaller islet size (Figure 4e,f) and β-cell mass (Figure 4h,i) were observed in HCR mice as compared to HFD mice. Furthermore, double immunofluorescence staining for CC3 and insulin on pancreas tissue sections indicated increased β-cell apoptosis in HCR group (Figure 5a,b).

**Figure 6:**
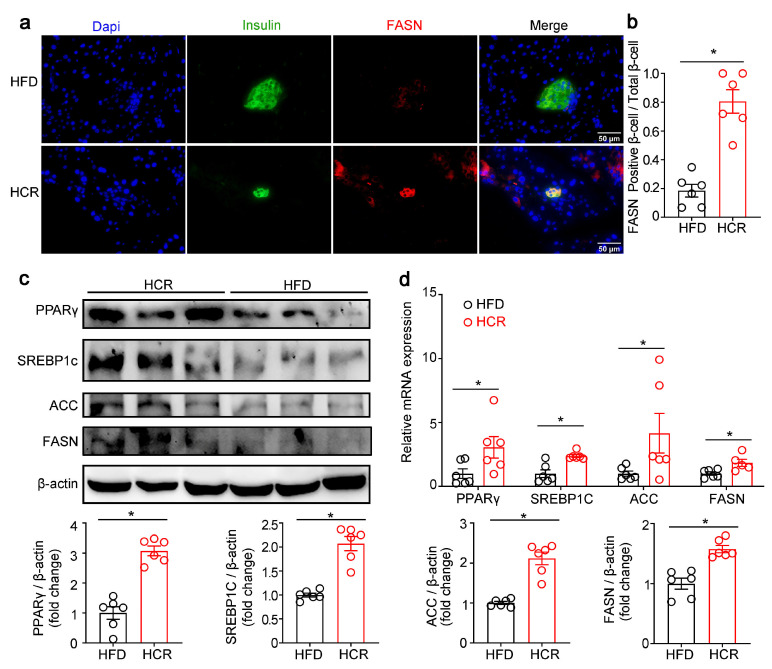
CR stimulated the lipogenic gene expression in pancreas of high-fat diet-fed mice. (**a**) Representative immunofluorescence images of fatty acid synthase (FASN, red) and insulin (green). (**b**) Quantification of FASN-positive β-cells/total β-cells. (**c**) Representative Western blot from HFD or HCR mouse pancreas. PPARγ, SREBP1c, ACC, and FASN were measured. Quantification of PPARγ, SREBP1c, ACC, and FASN. (**d**) PPARγ, SREBP1c, ACC, and FASN mRNA levels. The results were expressed as mean values ± SEM. (six mice per group), * *p* < 0.05 vs. HFD mice.

We next examined whether CR also activated lipogenic gene expression in the pancreas of HFD-fed mice. Immunofluorescence staining of FASN and insulin showed that FASN expression was markedly higher in the β-cell of HCR mice than that of HFD mice (Figure 6a,b). RT-qPCR and Western blot also showed upregulation of SREBP1c, PPARγ, FASN and ACC expression in the pancreas of HCR mice (Figure 6c,d).

### 3.3. Effects of RYGB on the Islet Morphology in Fatty Diet-Induced Diabetic Mice

**Figure 7:**
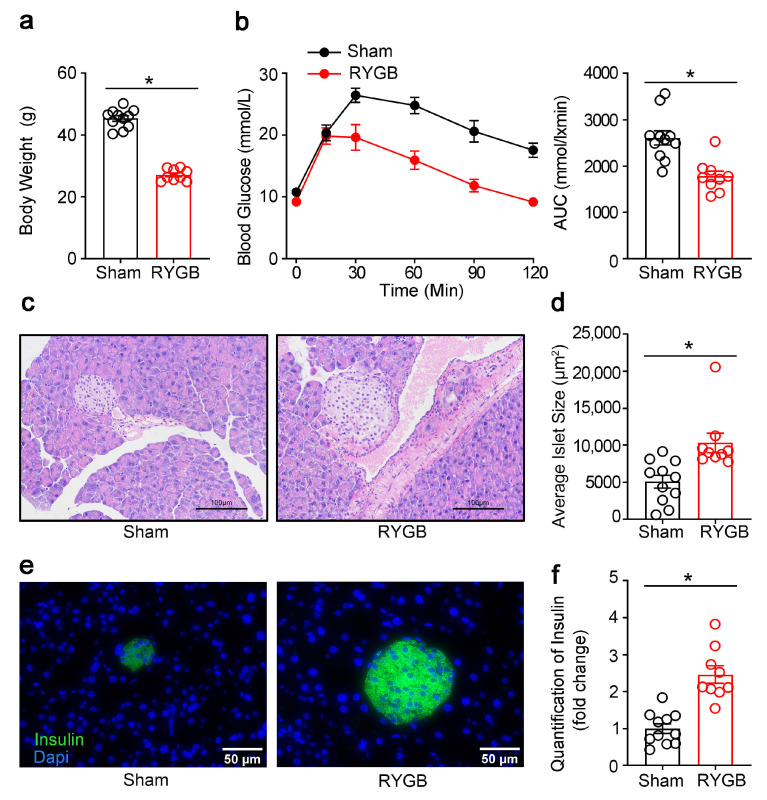
RYGB increased β-cell area in diet-induced diabetic mice. (**a**) Body weight of RYGB-treated diabetic mice. (**b**) Intraperitoneal glucose tolerance test. (**c**) HE staining of pancreatic sections. (**d**) Quantification of islet size. (**e**) Immunofluorescence staining for insulin (green) in the pancreas from the sham-operated or RYGB mice. (**f**) Quantification of insulin. The results were expressed as mean values ± SEM. (*n* = 11 for sham, *n* = 9 for RYGB), * *p* < 0.05 vs. sham-operated animals.

RYGB is an effective means for obesity and T2DM and has been found to have profound influence on glucose metabolism. We next investigated whether RYGB also affected β-cell mass and function. As shown in Figure 7, RYGB markedly decreased the body weight (Figure 7a) and improved the glucose intolerance induced by the high-fat diet (Figure 7b). Unlike CR in NCD and HFD, which led to smaller islet size and decreased β-cell mass, RYGB provoked a larger islet size (Figure 7c,d) and β-cell area (Figure 7e,f).

**Figure 8:**
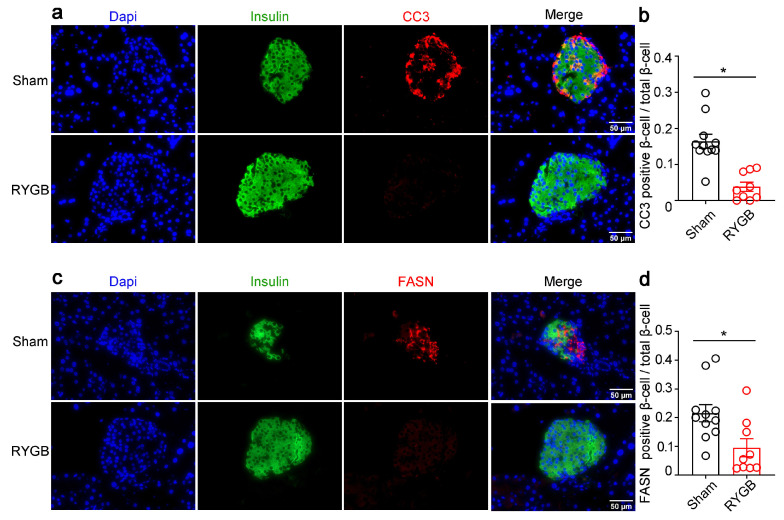
RYGB stimulated apoptosis of β-cells but decreased the expression of lipid synthesis genes. (**a**,**c**) Representative immunofluorescence images of the RYGB and sham-operated mice for cleaved caspase-3 (CC3, red), fatty acid synthase (FASN, red), and insulin (green). (**b**) Quantification of CC3-positive β-cells/total β-cells (β-cell apoptosis frequency). (**d**) Quantification of FASN-positive β-cells/total β-cells. The results were expressed as mean values ± SEM. (*n* = 11 for sham, *n* = 9 for RYGB), * *p* < 0.05 vs. sham mice.

In concordance with the increased β-cell after RYGB, decreased β-cells apoptosis was observed in RYGB mice, as indicated by immunofluorescence staining of CC3 and insulin (Figure 8a,b). Moreover, immunofluorescence staining for fatty acid synthase (FASN) showed a decline in the expression of FASN in the β-cells in RYGB mice (Figure 8c,d), suggesting a downregulation of lipogenic pathway in β-cells after RYGB.

## 4. Discussion

The major finding in the current investigation is that the alterations in islet morphology and function after both CR and RYGB may be related to pancreatic lipid. Adequate intracellular lipid is essential for β-cell function in the context of energy deprivation. CR stimulates the pancreatic endogenous lipid synthesis and β-cell apoptosis and also decreases β-cell area and insulin production in both normal and diabetic obese mice. RYGB inhibits the lipogenic gene expression and decreases the apoptosis of β-cells in diabetic mice.

Several studies have indicated that changes in β-cell mass are associated with obesity and diabetes. β-cell mass in subjects with obesity is considered to first increase, then decline with T2DM progression [7,8,34]. It has been reported that many factors such as lipotoxicity, oxidative stress, proinflammatory cytokines, glucotoxicity, and islet cell amyloid deposition are involved in β-cell apoptosis in T2DM [11,12,13,14]. Prevention of the loss of β-cell mass in T2DM is crucial to the long-term treatment of this disease [35]. Dietary restriction is employed as a first-line therapy option for T2DM. CR improves insulin resistance and glucose intolerance, thereby improving T2DM [36]. It is also believed that CR improves glucose metabolism by changing the β-cell mass [17]. There are conflicting reports about the effects of CR on β-cell mass. This may be related to the duration and the degree of CR, as well as the animal model. Sheng et al. demonstrated that long-term CR, such as for a period of 3 months (0.1 g/g body weight/day), reduced β-cell dedifferentiation and ameliorated β-cell function in db/db mice [37]. Kanda et al. also reported that 6 weeks of CR increased β-cell mass, stimulated β-cell proliferation and differentiation, and inhibited apoptosis and stress, which was attributed to the upregulation of ERK-1, Nkx2-2, Nkx6-1, NeuroD, and Pdx-1, as well as the downregulation of caspase-activated DNase (CAD) in db/db mice [17]. However, Gao et al. demonstrated that 3 weeks of moderate (40%) CR decreased the supranormal islet size, improved hyperinsulinemia and insulin sensitivity, and completely restored glucose homeostasis via the upregulation of β-cell autophagy in DIO mice [38]. Nevertheless, the underlying mechanisms involved in the effects of CR on β-cell morphology and function have not been fully clarified. Under basal circumstances, β-cells do not express death receptor Fas/CD95 (Fas). However, during diabetes progression, many factors, such as amylin and IL-1β, induce upregulation of Fas, mediating β-cell apoptosis [14,39,40]. In the current study, we found that CR increased the expression of CC3 in islets, leading to the apoptosis of β-cells. CR decreased the β-cell area via increasing β-cell apoptosis in both NCD-fed and HFD-fed mice. Lower insulin secretion is needed to maintain glucose homeostasis in the context of energy deprivation. Loss of β-cells may contribute to the decline in insulin synthesis and secretion. Our data also demonstrate that, in spite of being fed with the high-fat diet, a 30% reduction in food intake maintained a normal body weight and blood sugar. However, limitations exist in the study design. Restriction was for 4 weeks in NCR, but 8 weeks in HCR. These might cause greater effect of CR on apoptosis in HCR than NCR.

RYGB is an effective therapy for obesity and T2DM [16]. The improvement in hyperglycemia after RYGB occurs within days after the operation. RYGB was found to increase the β-cell mass in rats [21]. The activation of glucagon-like peptide 1 (GLP-1) and its signaling pathway and the inhibition of the NLRP3 inflammasome in islets may contribute to improved glucose homeostasis after RYGB [41,42]. Our study demonstrated that the increased islet insulin content after RYGB may be due to the decreased apoptosis of β-cells. Decreased apoptosis provides an advantage to β-cell growth and survival.

During the chronic intake of more energy than is required every day, excess energy is converted to fat in the liver and transferred to peripheral tissues for storage, which will increase fat delivery to all tissues and the pancreatic islets to take up fat avidly [43,44]. The excess fatty acid uptake brings about dysfunction, apoptosis, and dedifferentiation of β-cells, eventually triggering T2DM [12,29,30]. On the other hand, short-term exposure of β-cells to FFA is capable of enhancing GSIS [26,45]. It is known that a temporary increased plasma FFA level is one of the hallmarks of the fasted state, after infusion of the antilipolytic agent, decreased plasma lipid essentially abolish GSIS in both fasted rats and humans. Thus, an adequate supply of FFA in the fasting state is critical for the normal functioning of islet β-cells [25,26]. Our in vivo data demonstrate that the expression of lipogenic genes in the pancreas was increased rather than decreased under the condition of chronic energy deficiency caused by CR. Up-regulation of lipogenic gene expression increases the rate of β-cell apoptosis and decreases the β-cell area and insulin secretions, thus maintaining blood glucose homeostasis during energy deficiency.

Steven et al. determined that RYGB reduced pancreatic triglycerides in T2DM [46]. We further investigated the molecular mechanism underlying the decrease in pancreatic lipid accumulation after RYGB. Our data show that the decline in FASN expression in β-cells after RYGB may help reduce lipid accumulation in β-cells and improve β-cell function in the context of energy surplus, thereby alleviating T2DM.

## 5. Conclusions

In conclusion, this study examined β-cell apoptosis and the expression of lipogenic genes in the pancreas after both CR and RYGB. The alterations in the β-cell mass and insulin secretion after both CR and RYGB may be related to pancreatic fatty acid. Our study provides new insights into the pathogenesis of diabetes and potential therapeutic approaches.

## Figures and Tables

**Table 1:** List and sequences of primers used in the RT-PCR experiments.

	Upstream Primer (5′-3′)	Downstream Primer (5′-3′)	Accession Number(s)
*PPARγ*	TCAGCTCTGTGGACCTCTCC	ACCCTTGCATCCTTCACAAG	XM_017321456.3
*SREBP1c*	GGAGCCATGGATTGCACATT	GGAAGTCACTGTCTTGGTTGTTGA	XM_006532716.4
*FASN*	TGGGTTCTAGCCAGCAGAGT	ACCACCAGAGACCGTTATGC	NM_007988.3
*ACC*	TGGTCGTGACTGCTCTGTGC	GTAGCCGAGGGTTCAGTTCC	XM_006531956.3
*insulin*	CCTGGTGGAGGCTCTCTACCT	CAGAGGGGTAGGCTGGGTAGT	NM_001185084.2
*β-actin*	CCACAGCTGAGAGGGAAATC	AAGGAAGGCTGGAAAAGAGC	NM_007393.5

## Data Availability

All data are included in the article.

## Abbreviations

CR	Caloric restriction
RYGB	Roux-en-Y gastric bypass
SREBP1c	Sterol regulatory element-binding protein-1c
Fas	Death receptor Fas/CD95
FASN	Fatty acid synthase
ACC	Acetyl-CoA carboxylase
FFA	Free fatty acid
TG	Triglyceride
CC3	Cleaved caspase-3
GSIS	Glucose-stimulated insulin secretion
